# Conflict processing networks: A directional analysis of stimulus-response compatibilities using MEG

**DOI:** 10.1371/journal.pone.0247408

**Published:** 2021-02-25

**Authors:** Jessica Rosenberg, Qunxi Dong, Esther Florin, Praveen Sripad, Frank Boers, Martina Reske, N. Jon Shah, Jürgen Dammers

**Affiliations:** 1 Institute of Neuroscience and Medicine, INM-4, Forschungszentrum Jülich, Jülich, Germany; 2 Department of Neurology, RWTH Aachen University, Aachen, Germany; 3 JARA-Brain, Translational Medicine, Aachen, Germany; 4 Institute of Neuroscience and Medicine, INM-11, JARA, Forschungszentrum Jülich, Jülich, Germany; 5 Ubiquitous Awareness and Intelligent Solutions Lab, Lanzhou University, Lanzhou, China; 6 Institute of Clinical Neuroscience and Medical Psychology, Medical Faculty, Heinrich-Heine University Düsseldorf, Düsseldorf, Germany; Division of neurorehabilitation, SWITZERLAND

## Abstract

The suppression of distracting information in order to focus on an actual cognitive goal is a key feature of executive functions. The use of brain imaging methods to investigate the underlying neurobiological brain activations that occur during conflict processing have demonstrated a strong involvement of the fronto-parietal attention network (FPAN). Surprisingly, the directional interconnections, their time courses and activations at different frequency bands remain to be elucidated, and thus, this constitutes the focus of this study. The shared information flow between brain areas of the FPAN is provided for frequency bands ranging from the theta to the lower gamma band (4–40 Hz). We employed an adaptation of the Simon task utilizing Magnetoencephalography (MEG). Granger causality was applied to investigate interconnections between the active brain regions, as well as their directionality. Following stimulus onset, the middle frontal precentral cortex and superior parietal cortex were significantly activated during conflict processing in a time window of between 300 to 600ms. Important differences in causality were found across frequency bands between processing of conflicting stimuli in the left as compared to the right visual hemifield. The exchange of information from and to the FPAN was most prominent in the beta band. Moreover, the anterior cingulate cortex and the anterior insula represented key areas for conflict monitoring, either by receiving input from other areas of the FPAN or by generating output themselves. This indicates that the salience network is at least partly involved in processing conflict information. The present study provides detailed insights into the underlying neural mechanisms of the FPAN, especially regarding its temporal characteristics and directional interconnections.

## Introduction

It is widely accepted that human knowledge relating to the world around us is generally represented as schemas in our long-term memory [1]. Daily information is processed against the backdrop of our existing schemas [1]. Information that conflicts with these schemas leads to a goal-directed suppression of potential inappropriate responses [2]. In more detail, the ability to suppress inappropriate responses is required during situations in which the prevailing automatic response is inadequate or contradictory to the actual goal. This is referred to as conflict processing, or more generally termed, cognitive control [3]. Various experimental paradigms designed to study conflict processing have demonstrated that the presentation of a lateralised stimulus facilitates a congruent response with the ipsilateral hand [4–7]. In contrast, incongruent responses with the contralateral hand require the inhibition of the prevailing response and a reorientation to the opposite side, resulting in significantly longer reaction times [8]. The ability to suppress inappropriate responses is a key feature of executive functions and is essential for the adaptive control of everyday actions [9].

The use of brain imaging methods, such as functional magnetic resonance imaging (fMRI), to investigate the underlying neurobiological brain activations that occur during conflict processing have revealed the fronto-parietal attention network (FPAN, for a review, see Ptak (2012) [10]) to be the main area of activation. Interestingly, the FPAN has been reported to be influenced by aging [11], and its impairments seem to be associated with mental illnesses such as schizophrenia [12] and attention-deficit hyperactivity disorder [13]. In addition, postcentral gyri (PoC) activation was revealed during a motion-based Simon task [14], whereas an increase of precentral PrC activation was observed for response conflict and cognitive control during deceptive acts [15], respectively.

Causal interactions between the brain areas involved during conflict processing still have to be determined in more detail. For example, using fMRI, Fan, et al. [16] reported the left PoC to be linked to the left PrC, which in turn seems to be connected to the right PrC and the anterior cingulate cortex (ACC) during conflict processing. Furthermore, the same authors revealed a link between the ACC and the middle frontal cortex (MFC) [16]. However, as fMRI provides high spatial resolution but its temporal resolution is low, it cannot determine directional information of fast oscillating interactions. As a consequence, our knowledge of the underlying network of brain interactions related to conflict processing and the directionality of the temporal dynamics of the connections, which suggest causal relationships, remains incomplete.

**Fig 1:**
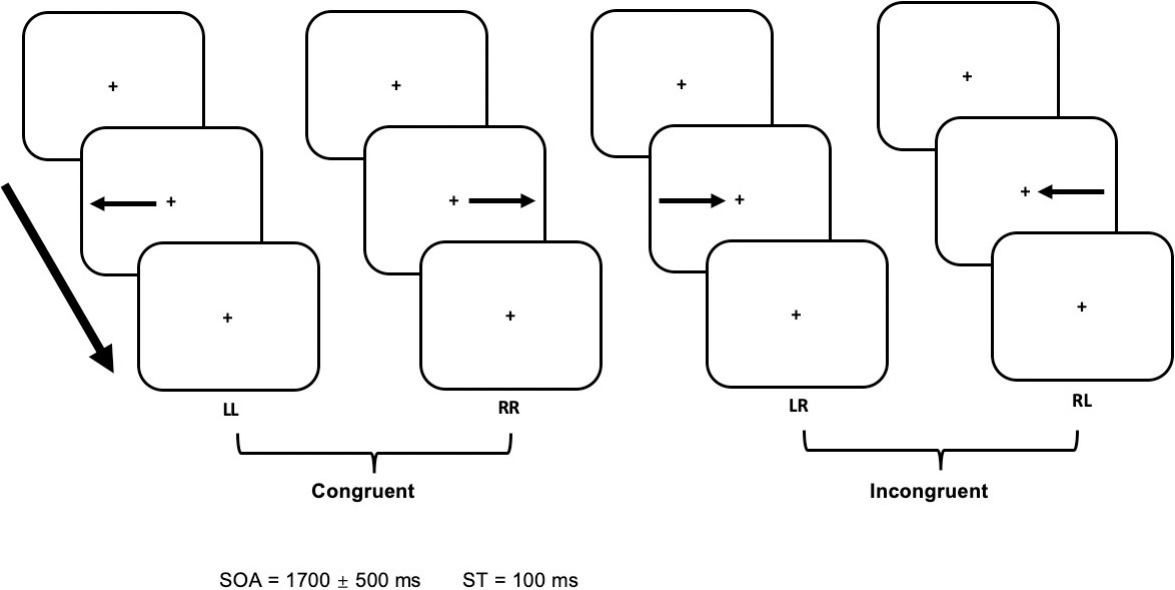
Stimuli design. During congruent conditions (LL, RR) the arrow was placed at the same side of the screen as where the arrow was pointing to, while during incongruent conditions (LR, RL), the placement of the arrow was opposite to its pointing direction. In each of the four different conditions (LL, LR, RR, RL) the first character denoted the direction of the arrow, while the second character indicated the ipsi- or contralateral side of the key-pressing finger. Subjects were asked to indicate the direction of the arrow utilising a left or right key press, respectively. The stimulus onset asynchrony (SOA) and stimulus duration are indicated at the bottom.

In contrast to fMRI, electroencephalography (EEG) can reflect the fast-oscillatory transfer of information between brain regions. Alterations in mid-frontal theta (4–8 Hz) and parietal alpha (8–12 Hz) power have been reported during conflict modulation [17, 18]. Pastötter and colleagues [19] focussed on the role of the ACC in conflict processing in particular, and their findings suggest that the ACC is linked to a theta power increase during conflict processing and inhibits the priming process, resulting in slower reaction times in congruent trials. Hereby, the involvement of cortical theta-band oscillations has been reported in top-down processes across broad (frontal) networks (for a review, see Cavanagh and Frank [20]). A study focussing on cognitive control, independent of awareness, mainly found synchronous oscillations in the medial prefrontal cortex [21]. The authors revealed that, during a Go/No-Go-Task (half of the No-Go Tasks were not consciously perceived), response errors enhanced oscillatory synchrony between the medial prefrontal and the occipital cortex. In Hesse et al. [22] the authors applied causality analysis on sensor level data recorded during a Stroop task [23] and demonstrated directed interactions from the posterior to the anterior cortical sites occurring 400 ms post Stroop stimulus. However, causality analysis conducted at the sensor level has proven to be less reliable [24, 25]. Moreover, EEG suffers from limited spatial resolution due to the inhomogeneous conductivity of different tissues, including the skull [26]. In addition, EEG signals are strongly influenced by large changes in electric conductivity between the brain, skull and scalp, creating marked distortion [24]. Therefore, we used magnetoencephalography (MEG), as the difference in the magnetic permeability across different tissues is negligible, thus causing fewer distortions and providing a better spatial accuracy compared to EEG [27, 28]. We aimed to identify the brain networks involved in conflict processing using an adaptation of the Simon task [29] (see Fig 1). Based on source-reconstructed MEG data, the cortical areas related to conflict processing were determined. For these brain regions, we then characterized the spatio-temporal dynamics, including the directional interactions, at different frequency bands. By doing this, we were able to identify active interconnections during conflict processing when a neural reorganisation is required.

## Materials and methods

### Participants

Fourteen right-handed healthy male volunteers (mean age 23.8, SD 4.1) participated in the present study. Subjects were recruited through internet alerts, newsletters and flyers and were financially compensated. The study was approved by the Ethics Committee of the Medical Faculty of the Rheinisch–Westfälische Technische Hochschule Aachen (RWTH Aachen University, EK157/10) and performed in accordance with the ethical standards of the institutional and national research committee and with the 1964 Helsinki declaration and its later amendments. All participants gave written informed consent before each examination. Only male subjects were included in order to reduce the number of confounding factors in the statistical analysis. All participants had either normal or corrected to normal vision. Handedness was assessed by the Edinburgh Handedness Inventory [30]. One subject was excluded because of unusually high noise in the data, hence the total number of subjects included for analysis was 13.

### Experimental procedure

The experimental paradigm was an adaptation of the Simon task [29]. Stimuli were generated using the Cambridge Research System (ViSaGe MKI; Cambridge Research System Ltd, Rochester, UK) and presented jitter-free, on a back-projection screen inside the magnetically shielded room. A DLP projector (Luxion LM-X25@60Hz, 1280x1024 Pixel) and a mirror system were used to project the stimuli. All subjects observed the stimulation in the supine position, facing the screen at a distance of 57 cm, resulting in a field of view of 40° × 30°. The visual stimuli consisted of arrows presented in black on a grey background. The arrows pointed either to the left or right side of a red fixation square placed in the centre of the screen. The arrows were presented on the midline of the screen (Fig 1). Each stimulus (left or right-pointing arrow) was presented for 100 ms in a pseudo-randomised order, i.e., with equal probabilities but mixed order, and with a stimulus onset asynchrony (SOA) ranging between 1200 ms and 2200 ms in steps of 16 ms.

Left and right-pointing arrows were presented either on the left or right side of the screen, resulting in two congruent and two incongruent conditions. During congruent conditions (LL, RR) the arrow was placed on the same side of the screen as where the arrow was pointing to (e.g., placement and pointing direction of the arrow was on the right side of the screen), while during incongruent conditions (LR, RL) the placement of the arrow was opposite to its pointing direction (Fig 1). Subjects were asked to indicate the direction of the arrow irrespectively of its placement. For example, for arrows pointing to the left, the subjects were asked to press a button using the left index finger, regardless of whether the arrow was presented on the right or left side of the screen. For arrows pointing to the right, the right index finger should be used. Within the task, a total of 320 stimuli were presented (80 for each condition) in a pseudo-randomised order.

### MEG data acquisition

Individual brain activity in response to the applied action intention paradigm was measured using MEG with a 4D-Neuroimaging whole-head magnetometer system equipped with 248 channels (MAGNES®-3600WH MEG). Neuromagnetic activity was continuously measured in the supine position with a sampling rate of 678.17 Hz and a bandwidth of 0 to 200 Hz. Eye movements and cardiac activity were monitored in synchrony with the MEG signals using electrooculography (EOG) and electrocardiography (ECG). In addition, five minutes of empty room data (i.e., recordings without a subject) were acquired before and after each MEG experiment. To combine neuromagnetic activity with structural information, individual 3D high-resolution T1-weighted magnetisation-prepared rapid acquisition gradient-echo (MPRAGE) anatomical scans (voxel size of 1 x 1 x 1 mm^3^) were acquired for each subject after the MEG experiment. A 3T Tim-Trio Siemens (Erlangen, Germany) magnetic resonance imaging (MRI) scanner was used for this.

### Behavioural data analysis

Statistical analysis was performed using SPSS (IBM SPSS Statistics 19; http://www.spss.com). Reaction time was measured from the moment the stimulus was presented until the participant made a response. The response onset referred to the digital output signal (light pulse) of the response device used. Reaction times (RTs) served as dependent variables. Significant differences in RTs between congruent and incongruent conditions were determined with analysis of the variance (ANOVA, *p* < 0.01). Effect sizes on reaction times were estimated using Cohen’s *d* and *f* parameters [31].

### MEG data analysis

For MEG data analysis, the MNE-Python software [32] was used. MEG signal channels were visually scanned for strong noise and artefact contaminations. In the case of such contaminations, the signals were replaced by a virtual channel using interpolated data from neighbouring channels [32]. The removal of environmental and power line noise was performed by subtraction of appropriate weighted reference signals from the MEG signals [33]. Ocular and cardiac activities were removed using OCARTA [34]. In addition, noise covariance matrices from empty-room recordings were computed for sub-sequent source localisation [35]. Only trials where the subject responded correctly were considered for further analysis. To avoid any bias from an unequal number of trials across conditions, the smallest number of correct trials found in all recordings across conditions for each subject was used for analysis.

### Source activity estimation and region of interest analysis

For anatomical localization, individual T1 weighted MRI scans were conducted on a 3T MAGNETOM Tim Trio scanner (Siemens, Erlangen, Germany) using standard gradients and a circular polarized phase array head coil. A magnetization prepared rapid gradient echo (MP-RAGE) sequence was acquired during the imaging session (TR = 2250 ms, TE = 3.03 ms, ST = 1 mm, FOV = 256 × 256 mm, voxel size = 1.0 × 1.0 × 1.0 mm^3^). To align neuromagnetic source activity estimations on structural information and for segmentation of the brain the FreeSurfer software package was used [36, 37]. For co-registration of the MRI and MEG data, and for estimation of the forward and inverse operator, the open-source toolbox MNE software was used [32]. The forward problem was solved using the boundary element method (BEM) with an average grid spacing of 3.1 mm. This method provided 10242 grid points for source analysis on the individual cortical surface, derived from individual MRI information in each hemisphere.

Source analysis using weighted minimum-norm estimates (wMNE [38]) was applied to both averaged and unaveraged data for the following reasons: i) source localisation was applied on averaged MEG signals to increase the signal-to-noise ratio (SNR) in order to enable identification of the active brain areas involved in processing the task; ii) for causality analysis, it was necessary to apply source reconstruction using unfiltered and unaveraged data [38–41].

ROIs were determined from filtered (1–45 Hz) and averaged source activity for each condition (LL, RR, LR, RL). These were acquired separately, using both stimulus onset averages (300 ms pre- and 300 post-stimulus) and response (button press) onset averages (200 ms before and 100 ms after response onset). This resulted in a set of 8 different averages for each subject. In order to identify active brain areas within the group of 13 subjects, a non-parametric, spatio-temporal cluster permutation, one sample t-test was performed to extract group-based statistical maps of spatial clusters with sustained (≥ 20 ms) temporal activations [42–45]. The 99.99^th^ percentile of the pre-stimulus activation (-300 ms– 0 ms) served as the significance threshold. The multiple comparison problem in this test was addressed by means of cluster-based permutations across space and time using 8191 (with 13 subjects, 2^13^–1 = 8191) repetitions. Significant spatio-temporal clusters with a p-value of *p* < 0.01 from each condition were merged and superimposed onto the “fsaverage” template brain [37]. To align the cluster activity to the anatomical structure, we used the Desikan-Killiany atlas [46, 47]. After delineation, small clusters, i.e., a cluster with a maximum vertex-to-vertex distance of 10 mm or durations shorter than 20 ms, were discarded from the analysis. The remaining clusters were used as ROIs in the following analysis.

For each experimental condition, all unfiltered MEG signal epochs from each condition (200 ms pre- and 800 ms post-stimulus) were used for single-trial (i.e., on single epochs) source localisation and were subsequently morphed onto the “fsaverage” brain space [40]. A representative source time course (rSTC) for each ROI was generated using the STC extraction tool provided by MNE-Python [32]. Principal component analysis (PCA) was applied to all vertex activations as defined by the cluster within each ROI. The mean scaled and first principal component, which takes the source orientation into account, was used for further analysis. All rSTCs were z-scored using the mean and standard deviation from the pre-stimulus interval (200 ms before stimulus onset) of each ROI and across all the trials for subsequent Granger causality analysis.

### Granger-based causality analysis

The post-stimulus time window ranging from 0–800 ms was used to determine the Granger-based causal interactions between ROIs. For this, it is necessary to fit a multivariate autoregressive (MVAR) to the rSTCs [48, 49]. The widely used Bayesian information criterion (BIC) was applied to determine the optimal model order [40, 50, 51]. After estimating the model order, the MVAR model was statistically evaluated [51–54]. Three statistical tests were applied to ensure the validity of the MVAR model: (1) The Durbin-Watson test was used to check if the MVAR residuals were white (*p* < 0.05). (2) The stability test was used to check that the VAR coefficients defined a covariance-stationary process [51], along with the stability index (SI) from the BSMART (version 0.5) Matlab toolbox. For SI being negative, the MVAR model was considered to be stable [49]. (3) The consistency test was used to evaluate the portion of data captured by the MVAR model. The MVAR model was deemed to have captured the data well if the consistency value was larger than 0.8 [49, 51]. Only MVAR models fulfilling all three statistical criteria were used for further analysis.

As this work focuses on the brain mechanisms of the incompatibility effect by comparing the differences between incongruent and congruent directional matrices, matrices were estimated according to the following pipeline:

1. On a single subject level generalised partial directed coherence (GPDC) was applied for Granger-based causality analysis to distinguish between direct and indirect connections [41, 52]. The method was applied to individual MVAR models (i.e., for each subject and condition separately) that passed all three criteria (Durbin-Watson test, stability test and the consistency test).
2. To evaluate the significance of the individual GPDC results of each condition, random surrogates were generated by shuffling the phases of the normalised rSTCs 1000 times [25, 55]. GPDC was then calculated for these surrogate data, and the 99.99^th^ percentile was used to define the significance threshold for GPDC.
3. GPDC results in the frequency range from 1 to 45 Hz were divided into 5 spectral matrices, corresponding to 5 frequency bands: theta (4–8 Hz), alpha (8–12 Hz), low beta (12–18 Hz), high beta (18–30 Hz), and low gamma (30–40 Hz). For each subject and condition, the GPDC spectral matrices from each ROI were averaged across frequency bins within each band.
4. The frequency-band averaged GPDC matrices were then binarized; values larger than the significance threshold were set to 1, and all other values were set to 0. Thus, for each subject and condition, five frequency binary *n*×*n* matrices were generated, with n being the number of ROIs.
5. A binomial statistical test with a critical alpha level of 0.01 was applied for group analysis to determine significant Granger-causal interactions between brain areas across subjects.

To investigate group differences in the dynamics of the (averaged) evoked responses during congruent and incongruent conditions, a non-parametric cluster permutation F-test was applied to the time courses of each ROI across subjects [42], using a significance level of *p* < 0.05.

## Results

### Incongruent stimuli cause longer reaction times as compared to congruent stimuli

**Fig 2:**
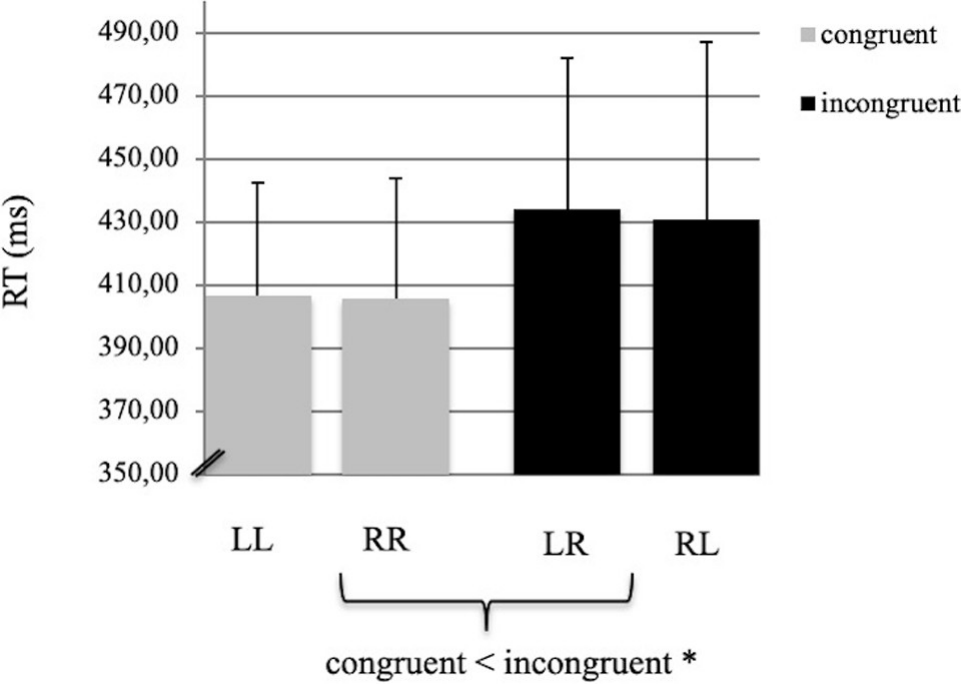
Mean reaction times. (RT, ms) during congruent (LL, RR) and incongruent (LR, RL) conditions. The error bars indicate the standard deviation. The asterisk marks a significant difference between the RTs of congruent versus incongruent conditions (*p* < 0.01).

The behavioural results revealed that subjects answered 91.2% of the trials correctly. No significant differences in error rates were found between congruent and incongruent trials (*p* > 0.20). The reaction times (RT) to incongruent stimuli (LR: mean = 433.79 ms, SD = 48.2 ms; RL: mean = 430.89 ms, SD = 55.9 ms) were significantly slower than to congruent stimuli (LL: mean = 406.6 ms, SD = 35.77 ms; RR: mean = 405.4 ms, SD = 38.42 ms; F(1,27) = 7.38, *p* < 0.01, Fig 2). Effect sizes for the ANOVA on reaction times (Cohen’s *d* and *f* parameters) were found to be 0.67 and 0.25, respectively. In each of the four different conditions (LL, LR, RR, RL), the first character denoted the placement of the arrow, while the second character indicated the ipsi- or contralateral side of the key-pressing finger. No differences were found between the left and right hand with respect to reaction times and error rates.

### Source localization revealed bi-hemispheric conflict processing

**Fig 3:**
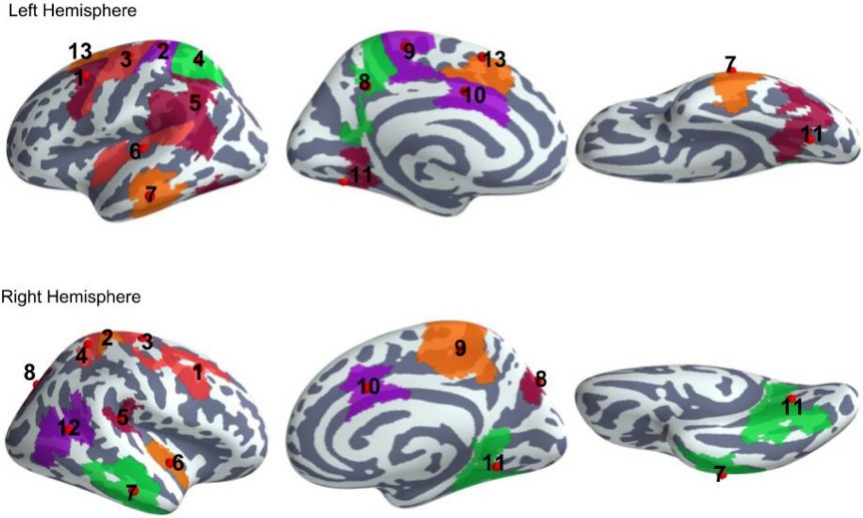
Map of regions of interest (ROIs) identified by a spatio-temporal cluster permutation test. Bilaterally activated regions were the MFC (caudal middle frontal cortex, 1), PoC (postcentral cortex, 2), PrC (precentral cortex, 3), SPC (superior parietal cortex, 4), SMC (supramarginal cortex, 5), AI (anterior insular cortex, 6), Tem (middle and inferior temporal regions, 7), Prec (precuneus and superior parietal regions, 8), Para (paracentral region, 9), ACC (anterior cingulate cortex, 10), and MVC (medial visual cortices, 11). In the right hemisphere, the IPC (inferior parietal cortex, 12) and in the left hemisphere the SFC (superior frontal cortex, 13) were significantly activated.

Fig 3 shows results from the source localisation of preprocessed MEG data. Spatio-temporal statistical testing revealed thirteen regions of interest (ROI). The centroids of the identified ROIs shown in Fig 3 are indicated with red dots. The corresponding anatomical region and centroid coordinates are listed in Table 1.

**Table 1:** ROI coordinates.

ROIs	Anatomical label	Cluster no.	Coordinates (lh)	Coordinates (rh)
**MFC**	Caudal middle frontal cortex	1	(-40, 10, 50)	(39, -81, 32)
**PoC**	Postcentral cortex	2	(-30, -30, 68)	(20, -31, 72)
**PrC**	Precentral cortex	3	(-36, -14, 64)	(36, -14, 64)
**SPC**	Superior parietal cortex	4	(-36, -51, 60)	(35, -45, 58)
**SMC**	Supramarginal cortex	5	(-57, -51, 30)	(42, -36, 22)
**AI**	Anterior insular cortex	6	(-44, -21, 4)	(41, -15, -11)
**Tem**	Middle and inferior temporal regions	7	(-59, -23, -18)	(63, -15, -19)
**Prec**	Precuneus and superior parietal regions	8	(-15, -50, 33)	(22, -83, 40)
**Para**	Paracentral region	9	(-6, -32, 56)	(9, -35, 53)
**ACC**	Anterior cingulate cortex	10	(-4, 2, 35)	(6, 13, 34)
**MVC**	Lingual and fusiform regions (medial visual cortices)	11	(-29, -68, -6)	(25, -56, -6)
**IPC**	Inferior parietal cortex	12	-	(53, -56, 12)
**SFC**	Superior frontal cortex	13	(-9, 14, 63)	-

The coordinates of ROI centroids identified by a spatio-temporal cluster permutation test (according to the “fsaverage” brain space atlas). Left hemisphere (lh), right hemisphere (rh).

**Fig 4:**
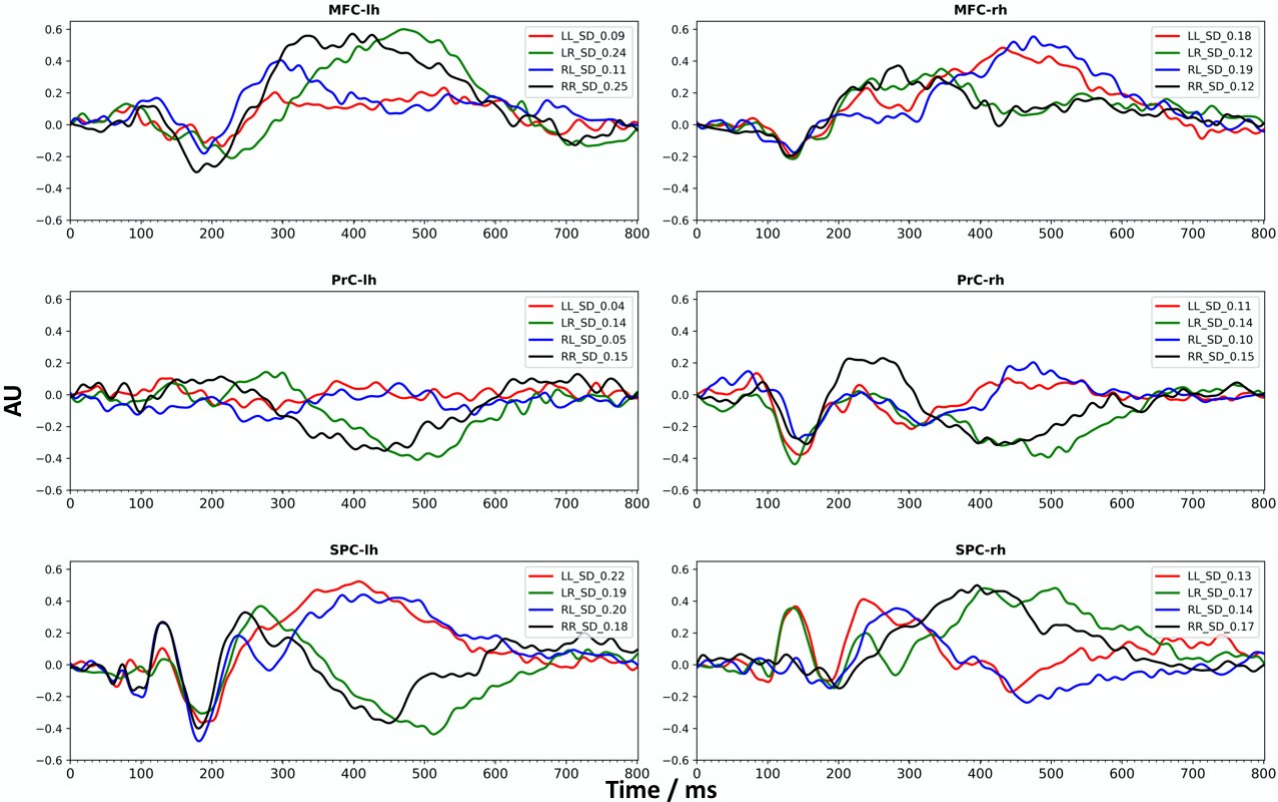
Time courses of significant bilateral activity. Group averaged time courses from single trail source analysis showing activity from the MFC (caudal middle frontal cortex), PrC (precentral cortex) and SPC (superior parietal cortex) for the left (lh) and right (rh) hemisphere and for each of the significant conditions of the conflict processing task (LL, RR, LR, RL) are presented. SD indicates the standard deviation; AU: arbitrary unit.

Time course analysis on evoked responses revealed that, during conflict processing in both conditions of conflict, the MFC, the PrC and the superior parietal cortex (SPC) were significantly activated bilaterally (Table 2 and Fig 4), i.e. during the processing of the arrow presented at the left-hand side of the screen (pointing to the right) and during the processing of the arrow presented at the right-hand side (pointing to the left).

**Table 2:** Time windows of significant activation during conflict processing (*p* < 0.05).

		lh		rh	
ROI	Cluster No.	RR vs. RL	LL vs. LR	RR vs. RL	LL vs. LR
MFC	1	331–524	353–552	417–600	405–541
PoC	2	368–538	389–611	n.s.	n.s.
PrC	3	330–524	362–608	412–550	393–564
SPC	4	334–580	328–631	358–608	358–566
SMC	5	n.s.	n.s.	384–545	325–533
AI	6	n.s.	305–535	n.s.	353–486
Prec	8	n.s.	n.s.	n.s.	411–591
Para	9	n.s.	244–364	373–521	356–561

Time in milliseconds, non-significant (n.s.), MFC (caudal middle frontal cortex), PoC (postcentral cortex), PrC (precentral cortex), SPC (superior parietal cortex), SMC (supramarginal cortex), AI (anterior insular cortex) Tem (middle and inferior temporal regions), Prec (precuneus and superior parietal regions), Para (paracentral region), ACC (anterior cingulate cortex), MVC (medial visual cortices), IPC (inferior parietal cortex), SFC (superior frontal cortex). Left hemisphere (lh), right hemisphere (rh).

The temporal dynamics are clearly different between congruent and incongruent stimuli (Table 2, Fig 4). For the MFC, an incongruent stimulus presented to the contralateral hemifield led to an earlier activation (200 ms– 350 ms) compared to an ipsilateral incongruent stimulus (around 400 ms– 600 ms). Moreover, an incongruent stimulus in the ipsilateral hemifield led to a positive activation between 400–600 ms, which was not the case for the contralateral hemifield. This effect is also seen for the right PrC. In contrast, for SPC an incongruent stimulus presented in the contralateral hemifield led to an increased (positive) activation from 400 ms– 600 ms and was decreased when presented in the ipsilateral hemifield. Peak latencies of the evoked M100 and M170 components estimated from group-averaged data are listed in the S1 Table.

The anterior insular cortex (AI), paracentral region, supramarginal cortex (SMC), PoC and precuneus (Prec) only revealed significant time courses during conflict processing in one hemisphere or in one of the incongruent conditions (see Table 3). The ACC, inferior part of the parietal cortex (IPC), medial visual cortices (MVC), superior frontal cortex (SFC) and the middle and inferior temporal regions (Tem) did not produce any significant time courses during conflict processing (see Table 2).

**Table 3:** Significant causal connections during conflict processing (*p* < 0.01).

Frequency band	LR vs. LL	RL vs. RR
theta	SPC (lh) → PoC (lh)	Para (lh) ↔ Para (rh)
(4–8 Hz)	Para (lh) ↔ Para (rh)	
	
	MVC (rh) → Tem (rh)	
alpha	SPC (lh) → PoC (lh)	AI (lh) → Tem (lh)
(8–12 Hz)	Para (lh) → Para (rh)	
	MVC (rh) → Tem (rh)	
	SMC (rh) → AI (rh)	
low beta	ACC (rh) → PrC (lh)	Para (lh) → PoC (lh)
(12–18 Hz)	Para (lh) → Para (rh)	AI (lh) → Tem (lh)
	IPC (rh) → SMC (rh)	SPC (rh) → AI (rh)
	SMC (rh) → AI (rh)	MFC (rh) → ACC (rh)
	Para (lh) → PoC (rh)	
high beta	ACC (rh) → MFC (lh)	AI (lh) → Tem (lh)
(18–30 Hz)	SMC (rh) → AI (rh)	SMC (rh) → IPC (rh)
	Para (lh) → PoC (rh)	
	Para (lh) → PrC (rh)	

MFC (caudal middle frontal cortex), PoC (postcentral cortex), PrC (precentral cortex), SPC (superior parietal cortex), SMC (supramarginal cortex), AI (anterior insular cortex), Tem (middle and inferior temporal regions), Prec (precuneus and superior parietal regions), Para (paracentral region), ACC (anterior cingulate cortex), MVC (medial visual cortices), IPC (inferior parietal cortex), SFC (superior frontal cortex).

### Directional interactions

The optimised model order for each multivariate autoregressive (MVAR) model ranged from 25 to 27. The MVAR models passed three statistical criteria (i.e., Durbin-Watson test, stability test and consistency test). For each subject and condition, generalized partial directed coherence (GPDC) was estimated for five different frequency bands (theta, alpha, low and high beta and low gamma). In this analysis, we focussed on the theta, alpha and beta bands as these frequency bands have been discussed to play a major role in conflict processing [17–20]. However, results from the gamma band analysis are provided in Supporting Information (S2 Table and S1 Fig).

**Fig 5:**
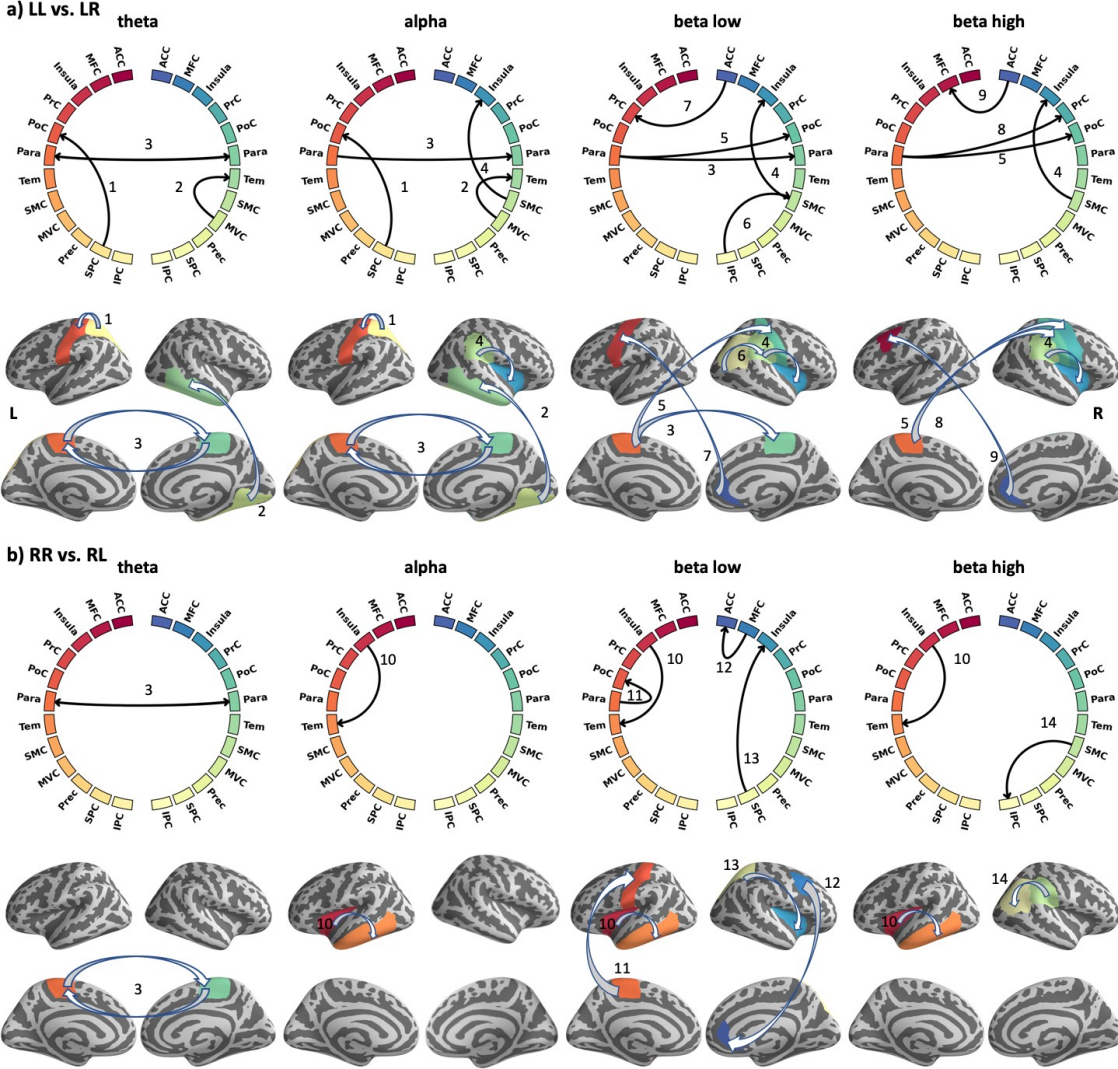
Causal information flow during conflict processing. Significant causal interactions (p < 0.01) are shown for both incongruent tasks, LL vs LR (a) and RR vs RL (a) at four different frequency bands (4–30 Hz). At each left and right part of the circle, ROI labels from the left and right hemispheres are shown respectively (cf. Table 3). The lines indicate significant connections, while arrows indicate the direction of information flow. The location of significant causal connections is mapped onto the surface of the brain for each frequency band. The same colour coding is used in the circles and brain plots.

Statistical group analysis (binominal test) revealed that two regions are connected if 10 subjects show directional information flow above the significance threshold (*p* < 0.01). Results are listed in Table 3 and visualised in Fig 5.

In contrast to congruent stimulations, processing of incongruent stimuli revealed significant differences in the information flow (Fig 5 and Table 3). These differences are evident not only across frequency bands, but also between the two contrasts LL vs. LR and RR vs. RL. Fig 5 indicates that more connections are found in higher frequency bands. In particular, the lower beta band seem to be highly involved in processing the incongruent tasks, while the theta band is less dominant. For example, the right ACC is recruited in both beta bands, while the MVC and Tem regions are only recruited in the theta to alpha range. The Para region shows connections in all frequency bands. When comparing the two contrasts, we found twice as much causal connections in the LL vs. LR contrast (16) as compared to the RR vs. RL contrast (8). Bi-directional connections between the ipsi- and contralateral side (i.e., across hemispheres) were found only in the theta band in the paracentral region (Fig 5).

## Discussion

The present study determined FPAN interconnections, their directionality and the temporal dynamics during conflict processing. As expected, subjects had significantly longer RTs for incongruent as compared to congruent, stimuli. Thus, the presentation of a lateralised stimulus facilitated a congruent response with the ipsilateral hand, whereas incongruent responses with the contralateral hand required the inhibition of the prevailing response and a reorientation to the opposite side. Therefore, the behavioural results of this study confirm that conflicting information requires a higher level of cognitive control for conflict resolution [56, 57].

Our findings further revealed that the MFC, the PrC and the SPC were significantly activated bilaterally during both conditions of conflict, i.e. during the processing of the arrow presented at the left-hand side of the screen (pointing to the right) and during the processing of the arrow presented at the right-hand side (pointing to the left) in a time range between 328 ms and 631 ms after stimulus onset. It is worth mentioning, that the duration of clustered activity and thus its start and end times, depends on the cluster threshold used. Our results are in line with findings reported by Hesse et al. [22] who demonstrated that conflict processing during the Stroop task occurred at around 400ms post Stroop stimulus. Notably, they conducted their causality analysis at the sensor level, which has been proven to be less reliable [24, 25, 58]. In contrast, Astolfi et al. [59, 60] reconstructed EEG data into the source space and constructed a directional network of the Stroop task at 12–29 Hz, revealing that the ACC is modulated by a broadly defined cingulate area and the dorsolateral PFC. However, the study focused on the evaluation of the estimators and only investigated 5 subjects. Moreover, it has been reported that the MFC, the PrC and the SPC play a major role in the FPAN during the resolution of conflict induced by incongruence between the spatial position of the stimulus and the required response hand [17, 61]. Our results corroborate that the ACC and AI are included in processing incongruent tasks. As illustrated in Fig 5, both the MFC and PrC share information with the right ACC in the beta band. The anterior insula (AI) receives information from the SMC and SPC. The exchange of information within the FPAN including the MFC, PrC, SPC, ACC and AI are processed at frequencies ranging from alpha to the higher beta band.

### Directional interaction within the FPAN

The present study provides a detailed description of the directional interactions of the FPAN. The different connectivity pattern we observed between the two contrasts LL vs LR and RR vs. RL is surprising, but may be caused by the small sample size and the significance threshold applied.

Notably, it was possible to reveal the interconnections that are particularly active during the neuronal reorganisation following the presentation of conflicting information on the high-beta band. Importantly, the ACC and the AI, which belong to the salience network, were also recruited in processing the conflict. This indicates that the salience network is at least partly involved in processing conflict information. In line with this finding, it has been reported that they play a major role in perceiving and monitoring conflicts [62–64]. Previous studies using fMRI to investigate conflict processing have reported that the ventrolateral prefrontal cortex (PFC) is strongly co-activated with the AI [65, 66], especially in tasks requiring inhibition of a response [63, 66]. Cai et al. [65] reported causal influences from the AI to the right posterior parietal cortex (PPC), from the dorsal ACC to the right PPC, and from the left PPC to the left dorsolateral PFC during conflict processing. The right dorsal ACC is reported to be interconnected with the right pre-motor cortex [64, 67], whereas the left dorsal ACC is influenced by the AI [65] and is interconnected to the left dorsolateral PFC [62]. The link between the left ACC and the left AI seems to represent the transformation and the neural reorganisation following the appearance of a conflicting stimulus. Similarly, it was determined that the right AI received input from the right SPC in the low beta band, while the left SPC generated input for the left PoC. FMRI studies reported the left PoC to be linked to the left PrC, which in turn seems to be connected to the right PrC and the ACC during conflict processing [16]. Interestingly, the present study supplements this information by showing that the left PrC is influenced by the right and left ACC in the low beta band. Additionally, and confirming our results, the ACC has been reported to be linked to the MFC [16]. A notable finding from the present study is that the left ACC receives input from the left AI in the low gamma band and therefore provides additional information about conflict processing in the frequency domain (S2 Table and S1 Fig).

In summary, the present study reveals that the right ACC is influenced by the right MFC in the low beta band while the left MFC is influenced by the right ACC in the higher beta band. To our knowledge, the role of the MFC in conflict processing remains actively debated (e.g., review by Ridderinkhof et al., 2004 [68]). Thus, our results regarding bidirectional activations at different frequency bands between the two anatomical regions could indicate an interaction in related conflict-processing-functions such as reorienting attention or error monitoring. Advantageously, the present study depicts, in detail, at which frequency bands the brain areas represented in the FPAN share directional information. It should be noted that the AI is active in many frequency bands, with the exception of the theta band. Interestingly, the AI was bilaterally activated during a time window ranging from 305ms to 535ms in the left hemisphere and from 353ms– 486ms in the right hemisphere. However, this was only the case when a conflict task was induced in the left visual hemifield.

### Limitations of the study

A limitation of the study is that only male subjects were included. This was done to eliminate potential hormonal effects and to avoid adding another level of statistical complexity because the sample was originally taken from a study focussing on circadian rhythms. Hence, our results speak for males only and need to be replicated in females. Moreover, the analysis was performed on MEG data recorded in 13 subjects only. Thus, our results must be interpreted in the context of a limited sample size. Furthermore, the present study focuses on the long-lasting effects of conflict processing and also includes event-related activity. Despite determining the nature of the FPAN connections during conflict processes, the question of how far the evoked activity covers connectivity from other faster cognitive processes, which were not captured by the actual Granger causality analysis, remains. Therefore, one area for future research would be to add another level of complexity to our study design by looking at shorter time windows after stimulus onset in a sliding window approach in more detail.

In conclusion, the present study provides detailed insights into the underlying neural mechanisms of the FPAN, especially regarding its temporal characteristics and directional interconnections. Therefore, our findings have strong implications for future research focusing on distortions of the FPAN, as it has been reported that the network is influenced by ageing and appears to be associated with mental illnesses such as schizophrenia and attention-deficit hyperactivity disorder. It is anticipated that our results will have an impact on future research on distortions of the FPAN.

## Supporting information

S1 Table Peak latencies. (XLSX)

S2 Table Causal connections in the lower gamma band. (XLSX)

S1 Fig Causal information in the lower gamma band. Information flow during conflict processing in the frequency range from 30 to 40 Hz. The link between the left ACC and the left PrC seem to reflect a relatively high level of workload during processing of conflicts. The information flow from and to the ACC and AI were observed for most of the frequency bands.
(TIF)
