# Supplementary figures and images for: Conflict processing networks: A directional analysis of stimulus-response compatibilities using MEG
Source: PLoS One. 2021 Feb 25;16(2):e0247408. doi: 10.1371/journal.pone.0247408 (PMC7906351; doi:10.1371/journal.pone.0247408)

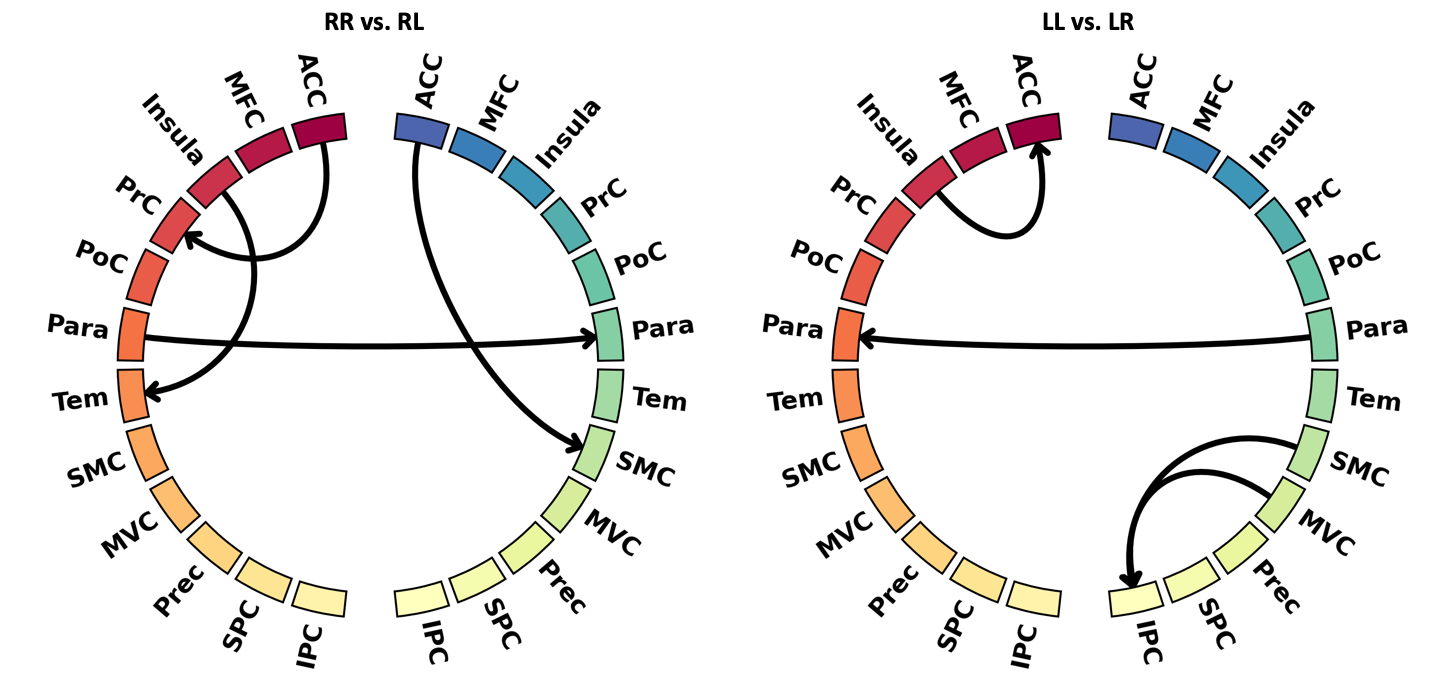

Supplement: S1 Fig — Information flow during conflict processing in the frequency range from 30 to 40 Hz. The link between the left ACC and the left PrC seem to reflect a relatively high level of workload during processing of conflicts. The information flow from and to the ACC and AI were observed for most of the frequency bands. (TIF) [file pone.0247408.s003.tif]
